# Resumption of anti‐programmed cell death 1 monotherapy for severe immune‐related adverse events experienced patient with renal cell carcinoma

**DOI:** 10.1002/iju5.12173

**Published:** 2020-06-25

**Authors:** Yoko Maegawa, Taigo Kato, Shinichiro Fukuhara, Hiroshi Kiuchi, Ryoichi Imamura, Motohide Uemura, Norio Nonomura, Kazutoshi Fujita

**Affiliations:** ^1^ Department of Urology Osaka University Graduate School of Medicine Osaka Japan; ^2^ Department of Urological Immuno‐oncology Osaka University Graduate School of Medicine Osaka Japan

**Keywords:** immune checkpoint inhibitor, immune‐related adverse event

## Abstract

**Introduction:**

Combined anti‐cytotoxic‐T‐lymphocyte antigen 4 and programmed cell death 1 blockade induced high rates of immune‐related adverse events in patients with renal cell carcinoma. However, the safety of reinitiating anti‐programmed cell death 1 monotherapy for patients who discontinued combination therapy due to immune‐related adverse events is largely unknown.

**Case presentation:**

We report the case of 74‐year‐old man who received combination therapy with anti‐cytotoxic‐T‐lymphocyte antigen 4 and programmed cell death 1 inhibitors for advanced renal cell carcinoma. After three cycles of combination therapy, he complained severe immune‐related adverse events including grade 3 nausea and anorexia, and grade 3 diarrhea, leading to discontinuation of the therapy. He started readministration of anti‐programmed cell death 1 monotherapy at 41 weeks after discontinuation due to the new lung metastatic lesion. Importantly, he experienced only grade 1 diarrhea, which can be controlled with prednisolone.

**Conclusion:**

The readministration of anti‐programmed cell death 1 monotherapy with close monitoring can be an acceptable treatment even after discontinuation of combination therapy.

**Abbreviations & Acronyms**
CT: computed tomography
CTLA‐4: cytotoxic‐T‐lymphocyte antigen 4
ICI: immune checkpoint inhibitor
Ipi: ipilimumab
irAE: immune‐related adverse event
Nivo: nivolumab
PD‐1: programmed cell death 1
PSL: prednisolone
RCC: renal cell carcinoma

**Keynote message**

The safety of resuming anti‐PD‐1 monotherapy for patients who discontinued ICIs due to irAEs is still unknown. We report an instructive case with advanced RCC who carefully resumed anti‐PD‐1 inhibitor after discontinuation of dual CTLA‐4 and PD‐1 blockade due to severe irAEs.

## Introduction

ICIs have revolutionized the field of cancer in recent years and remarkably improved the prognosis of several types of cancer.^1^ As ICIs have been used over the years, a certain number of patients face discontinuation of ICIs due to severe and sometime life‐threatening irAEs.^2^ Especially, combination of CTLA‐4 and PD‐1 inhibitors, ipilimumab and nivolumab, was shown to increase the incidence of irAEs compared to that of either monotherapy despite of the high response rate.^3^, ^4^

Normally, patients with high‐grade irAEs require discontinuation of both ipilimumab and nivolumab with the administration of corticosteroids. After treatment‐free duration, physicians often need to choose secondary treatment in the face of progressive disease. Considering that most severe toxicities occur within 3 months after initiation of combination therapy, readministration of anti‐PD‐1 monotherapy can be the optional treatment to achieve durable disease control. However, the safety and efficacy of resuming anti‐PD‐1 monotherapy is still largely unknown.

Here, we report an instructive case with advanced RCC who carefully resumed anti‐PD‐1 inhibitor after discontinuation of dual CTLA‐4 and PD‐1 blockade due to severe irAEs.

## Case presentation

**Fig. 1:**
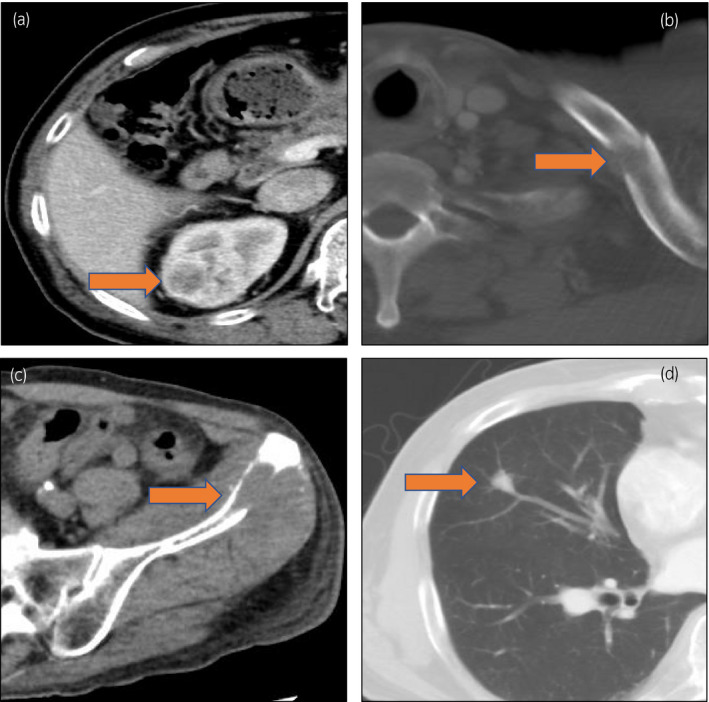
Abdominal and chest CT scan at the first diagnosis. Abdominal and chest CT shows the renal mass with early enhancement in right kidney with 35 mm diameter (a), osteolytic mass in the left clavicle (b) and left iliac crest (c), and small nodule in middle lobe of the right lung (d).

A 74‐year‐old man with no significant medical history presented with his left clavicular pain. A chest X‐ray showed a neoplastic fracture in the left clavicle. Abdominal and chest CT examination revealed the renal mass with early enhancement in right kidney with 35 mm diameter (Fig. 1). In addition, CT scan showed osteolytic mass in the left clavicle, left iliac crest, and small nodule in middle lobe of the right lung (Fig. 1). It is the basic policy of our institution to recommend radical nephrectomy for clinical stage T1 and T2, and subsequently he underwent laparoscopic radical nephrectomy. The pathological examination showed clear cell RCC (Fuhrman grade 2) with tumor invasion of perirenal fat, leading to the diagnosis of stage pT3aN0M1. With a diagnosis of left clavicle and iliac bone metastasis, the patient received the treatment with zoledronic acid 4 mg intravenously every 4 weeks.

According to the International Metastatic RCC Database Consortium score, the patient was classified as poor risk (<1 year from time of diagnosis to systemic therapy, Karnofsky Performance Status 70, low hemoglobin value) and began to receive nivolumab plus ipilimumab combination therapy for his multiple metastatic sites.

**Fig. 2:**
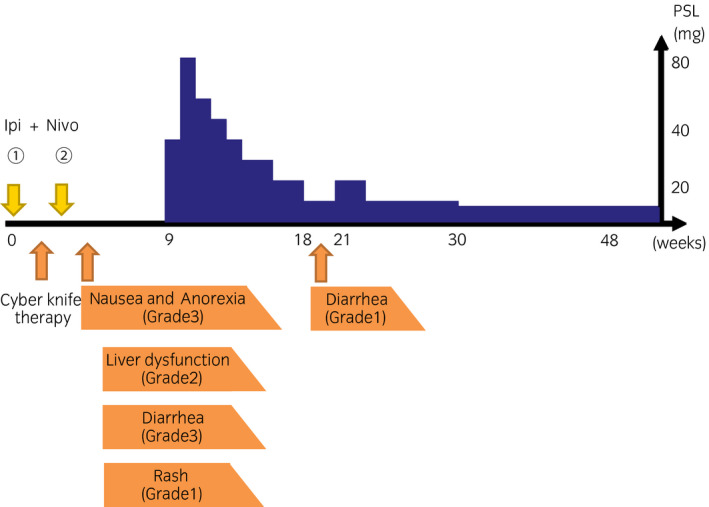
Clinical course of the case with severe irAEs induced by ipilimumab and nivolumab. After two cycles of combination therapy, the patient showed severe irAEs such as grade 3 nausea and anorexia, and grade 3 diarrhea. All symptoms gradually resolved within next 7 weeks after discontinuation of combination therapy and additional treatment of PSL.

**Fig. 3:**
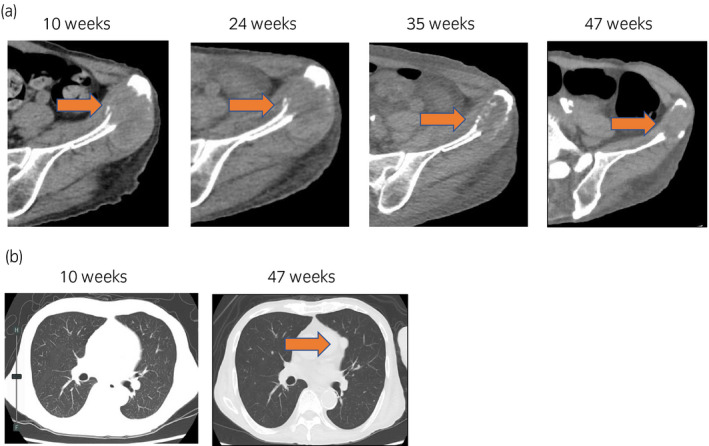
Sequential CT scans after discontinuation of combination therapy. (a) Abdominal CT scans show that the metastatic site of iliac bone maintained partial response for 30 weeks after the discontinuation of combination therapy without any additional therapy. After 41 weeks after discontinuation follow‐up, CT showed reduction of iliac bone metastatic sites. (b) Chest CT scan shows the new lung metastatic lesion at 41 weeks after discontinuation and the patient was diagnosed as progressive disease. The number of weeks indicates duration after the first initiation of combination therapy.

**Fig. 4:**
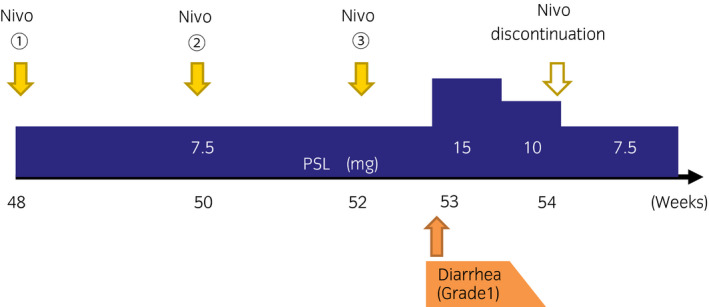
Clinical course after the readministration of nivolumab for progressive disease. The patient experienced only grade 1 diarrhea after readministration of nivolumab. The symptom promptly resolved with temporary increasing dose of PSL.

After one cycle of combination therapy, he felt listlessness of the right forearm and difficulty in speaking clearly. The brain magnetic resonance imaging showed a brain metastasis and cyber knife therapy was performed for brain metastasis (Fig. 2). In parallel with radiotherapy, the patient received the second course of the combination therapy. Two weeks later, he complained of grade 3 nausea and anorexia. He also experienced grade 3 diarrhea, grade 2 liver dysfunction, and grade 1 rash, leading to discontinuation of third course of combination therapy (Fig. 2). We suspected severe irAEs due to various symptoms, but we were unable to exclude the possibility of bacterial enteritis and started the administration of 40 mg (1 mg/kg per day) PSL for the patient. With the negative test result of fecal culture, the dose of PSL was increased to 80 mg and irAEs gradually improved within next 7 weeks. Finally, the dose of PSL was tapered to 7.5 mg. Metastatic sites maintained partial response for 30 weeks after the discontinuation of ICIs without any additional therapy (Fig. 3a). However, follow‐up CT examination showed the new lung metastatic lesion after 41 weeks after discontinuation (Fig. 3b) and we decided to start readministration of nivolumab monotherapy (Fig. 4). As a result, he experienced only grade 1 diarrhea after three cycles of nivolumab, which can be controlled with the temporary increasing dose of PSL. Importantly, any other irAEs such as nausea, anorexia, liver dysfunction, and rash did not appear after the introduction of nivolumab monotherapy.

Follow‐up CT scan at 12 weeks after the introduction of nivolumab showed the increase in lung and iliac bone metastasis, leading to withdrawal of nivolumab and subsequent axitinib therapy.

## Discussion

Immunotherapy targeting immune checkpoint is now the most attractive therapy in cancer field.^1^ Particularly, combination of ICIs expands the application to various types of cancer as the first‐line therapy because of the high response rate.^5^ At the same time, it is undeniable that a certain population of patients are forced to discontinue combination therapy because of severe irAEs. However, there is no conclusive evidence that determine which second‐line therapy is suitable in patients with progressive disease after discontinuation of ICIs.

In this study, we report a patient who experienced severe irAEs while on combination therapy of ipilimumab and nivolumab, and were cautiously reinitiated with anti‐PD‐1 monotherapy after certain duration of discontinuation. Interestingly, at the reinitiation phase with nivolumab, the patient experienced only grade 1 diarrhea. Pollack *et al*. reported almost 40% of 80 melanoma patients who discontinued combination therapy experienced recurrent or clinically significant distinct (de novo) irAEs with anti‐PD‐1 monotherapy reinitiation.^6^ They concluded that most of recurrent or distinct irAEs were low‐grade and manageable with corticosteroid treatment, while they had one case of grade 5 Steven–Johnson syndrome. In other study, the same or different irAEs occurred in 55% of patients with resuming anti‐PD‐1 or anti‐PD‐L1 therapy and were not found to be more severe than the first.^7^ Given that major irAEs (e.g. colitis, hypophysitis) of combination therapy are largely associated with ipilimumab at the first line of treatment, anti‐PD‐1 resumption with careful monitoring of irAEs may be one of the options to achieve further clinical response, although further cases are needed to judge the efficacy of anti‐PD‐1 resumption.

In conclusion, with intent to “cure” for cancer, the readministration of anti‐PD‐1 monotherapy may be considered as a durable and feasible option for RCC patients who discontinued combination therapy.

## Conflict of interest

The authors declare no conflict of interest.
